# Evaluating large language models for automated TNM staging from PET-CT reports: a multi-cancer comparative study

**DOI:** 10.3389/fdgth.2026.1741973

**Published:** 2026-03-04

**Authors:** Wen Xu, Lixiu Cao, Qijun Shen, Yanna Shan, Shushu Pan, Mei Ruan

**Affiliations:** 1 Department of Radiology, Hangzhou First People’s Hospital, Hangzhou, China; 2 Department of Nuclear Medicine Imaging, Tangshan People’s Hospital, Tangshan, China

**Keywords:** artificial intelligence, large language models, oncology, PET-CT, TNM staging

## Abstract

**Purpose:**

To evaluate three large language models (LLMs), including ChatGPT 5, ChatGPT 4o, and ChatGPT 3.5, in automating TNM staging from PET-CT reports across six cancer types, and to assess their clinical utility compared with junior radiologists.

**Materials and methods:**

PET-CT reports from 552 treatment-naive patients in two institutions with confirmed primary malignancies (lung, breast, liver, pancreatic, renal, and prostate cancer) were analyzed. Three ChatGPT-series LLMs and five junior radiologists independently performed TNM staging. Reference standards were established by two senior radiologists according to the 8th version of American Joint Committee on Cancer (AJCC) staging system. Performance was evaluated using accuracy rates. Intra-model agreement was assessed by repeating each model three times per report with identical prompts, and inter-model agreement was evaluated using Cohen's *κ* coefficients.

**Results:**

ChatGPT 5 achieved the highest overall accuracy (82.1%, 453/552), followed by ChatGPT 4o (74.3%, 410/552), both significantly outperforming ChatGPT 3.5 (59.6%, 329/552) and junior radiologists (77.0%, 425/552; *p* = 0.041 for ChatGPT 5 vs. junior radiologists). Accuracy varied by cancer type, with the highest performance in lung cancer staging (88.5%) and the lowest in pancreatic cancer (69.2%). Across TNM categories, all models achieved the best performance in T staging, followed by N staging, with M staging remaining the most challenging. ChatGPT 5 showed near-perfect intra-model agreement (*κ* = 0.96), while inter-model agreement ranged from moderate between ChatGPT 3.5 and 4o (*κ* = 0.58) to substantial between ChatGPT 5 and 4o (*κ* = 0.78). ChatGPT 5 processed cases markedly faster than junior radiologists (8.3 ± 3.2 vs. 92.5 ± 21.7 s per case; *p* < 0.001).

**Conclusion:**

Among the three LLMs, ChatGPT 5 demonstrated the highest accuracy, stability, and efficiency in automated TNM staging from PET-CT reports, achieving performance comparable to or slightly exceeding junior radiologists. Its advantages in T staging and lung cancer evaluation highlight its clinical utility as a potential decision-support tool.

## Introduction

The TNM staging system remains the cornerstone of cancer prognosis and treatment planning (1). Accurate staging guides therapeutic strategies, predicts outcomes, and informs multidisciplinary management. In oncology practice, staging derived from PET-CT is particularly important, as this modality combines functional and structural information into a single examination (1). However, translating narrative PET-CT reports into precise TNM categories is labor-intensive and subject to variability, especially among junior clinicians. Inconsistent staging can directly influence therapeutic recommendations, clinical trial eligibility, and longitudinal outcome assessment, making reproducibility an essential requirement in daily practice (2).

Large language models (LLMs) have emerged as promising tools for medical text interpretation (2–4). The ChatGPT family in particular has attracted attention for its ability to generate coherent responses, structure radiology reports, and even pass board-style examinations (5, 6). Beyond clinician-facing tasks, LLMs have also shown strong performance in answering patient care questions in oncology settings (7). Applications in protocol selection, multilingual report translation, and automated impression generation further illustrate the versatility of these systems within radiology (3, 4). Such progress suggests that LLMs may be capable of tackling more structured and reasoning-intensive tasks, including mapping free-text PET-CT reports to standardized oncologic staging systems. Importantly, successive iterations from ChatGPT 3.5 to ChatGPT 4o and most recently ChatGPT-5 have shown marked gains in reasoning, factual consistency, and efficiency, underscoring the need to reassess performance with each new generation (8–10).

Despite these advances, the role of LLMs in systematic oncologic staging remains poorly understood. Existing studies have generally addressed individual diagnostic questions or text summarization tasks, but systematic multi-organ TNM classification has not been thoroughly examined (5, 6, 11). Moreover, comparisons across different model generations are lacking, and head-to-head benchmarking against human readers remains scarce. Whether LLMs can provide reliable staging across cancers of different organ systems, and how their performance compares with that of junior radiologists, has yet to be established.

The aim of this study was therefore to evaluate the performance of ChatGPT 5, ChatGPT 4o, and ChatGPT 3.5 in automating TNM staging from PET-CT reports across six common cancer types. Reference standards were established by senior radiologists, and results were benchmarked against junior radiologists to assess accuracy, reproducibility, and efficiency in a clinically relevant setting.

## Materials and methods

### Patient cohort and data collection

**Figure 1:**
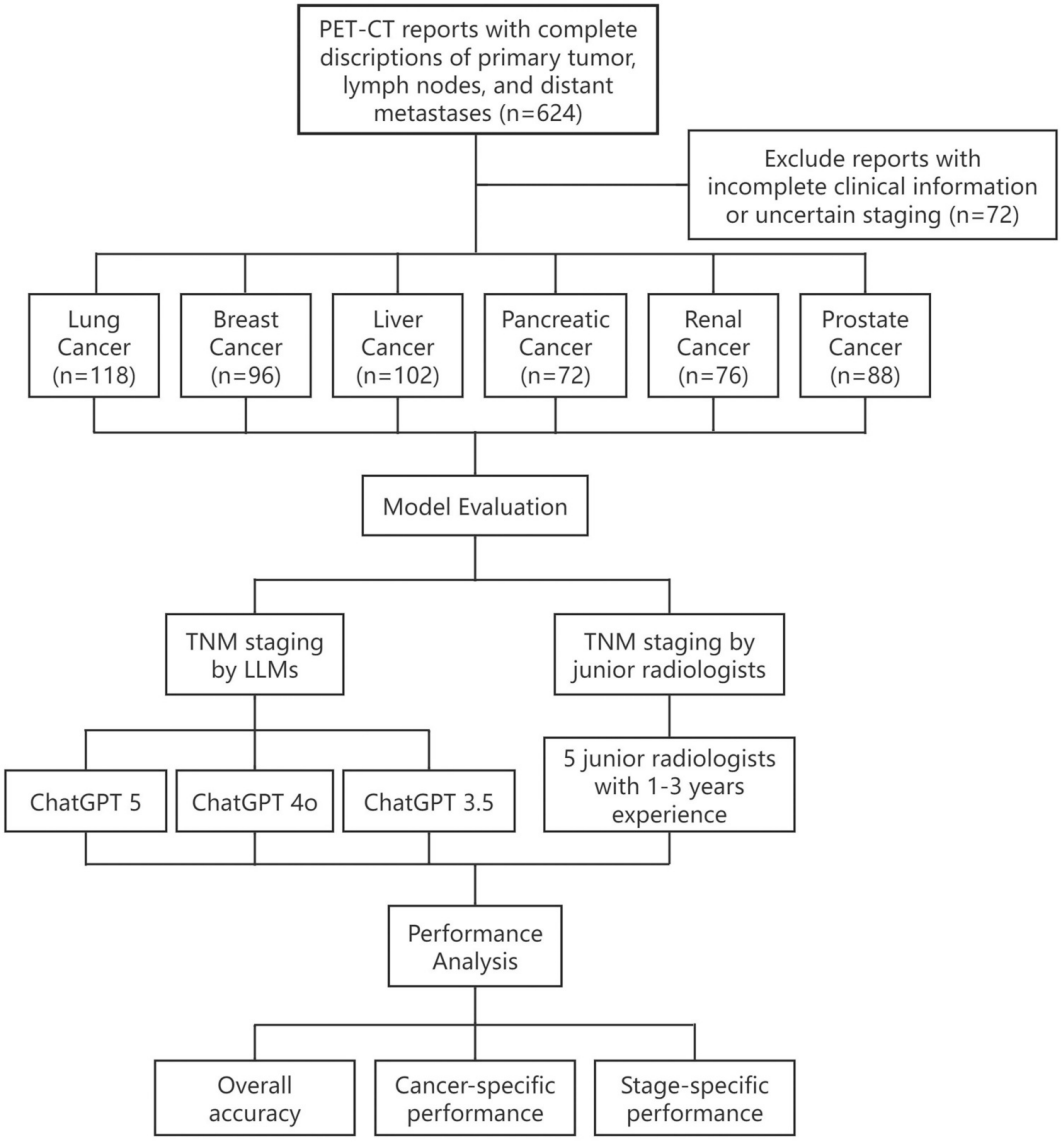
Flow diagram of patient selection and study design. PET-CT reports from 552 treatment-naive patients across six cancer types (lung, breast, liver, pancreatic, renal, and prostate) were retrospectively collected from two institutions. Reports were staged independently by three large language models (ChatGPT 5, ChatGPT 4o, and ChatGPT 3.5) and by junior radiologists, with senior radiologists providing the reference standard.

This retrospective study included 552 PET-CT reports from patients with histologically confirmed primary malignancies, collected between January 2020 and December 2024 from two tertiary institutions. Cancer types comprised lung (*n* = 118), breast (*n* = 96), liver (*n* = 102), pancreatic (*n* = 72), renal (*n* = 76), and prostate (*n* = 88) cancers (Figure 1).

Inclusion criteria were: (1) treatment-naive patients; (2) availability of complete PET-CT reports with diagnostic-quality images; and (3) availability of a high-confidence reference TNM label, defined as senior radiologist–adjudicated clinical TNM (cTNM) that was concordant with the final staging information documented in the medical record (including pathological staging when available). Reports with incomplete information or indeterminate staging were excluded.

### Report format and text preprocessing

The PET-CT reports followed an institutionally standardized template with fixed section headers. For model input, we extracted only the “Findings” and “Impression” sections and removed administrative headers/footers and any patient identifiers. No manual rewriting, normalization of terminology, or rule-based restructuring of the clinical content was performed. The extracted text was then translated into English and reviewed to ensure that clinical meaning was preserved. The final model input consisted of the concatenated Findings and Impression text for each case.

### Reference standard TNM staging

TNM staging was defined according to the 8th edition AJCC staging system. The reference standard was the consensus clinical TNM (cTNM) based on PET-CT, established by two senior radiologists (15 and 12 years of oncologic imaging experience) who independently reviewed the PET-CT images together with the corresponding report text. Clinical data were restricted to information necessary to apply the appropriate cancer-specific AJCC criteria and resolve staging ambiguities; no additional downstream pathologic upstaging information was used to assign TNM categories beyond what was supported by PET-CT findings. Discrepancies were resolved by consensus with a third radiologist (20 years of experience).

### Large language model evaluation

Three LLMs were evaluated: ChatGPT 5 (version: gpt-5; August 2025), ChatGPT 4o (version: gpt-4o-2024-11-20; November 2024), and ChatGPT 3.5 (version: gpt-3.5-turbo-0125; January 2024). All analyses were conducted during a fixed study period in August 2025 to ensure consistency of model performance and to minimize potential influence of model updates.

A standardized prompt was applied to all models:

“You are an expert radiologist. Based on the following PET-CT report, please determine the TNM stage according to the AJCC 8th edition criteria for [specific cancer type]. Provide separate assessments for T, N, and M with reasoning.”

All experiments were executed via the OpenAI API. For each model, we used identical prompts and report text inputs across runs, with parameters held constant to reduce sampling variability (temperature = 0; top_*p* = 1.0; presence_penalty = 0; frequency_penalty = 0; max_tokens left at the API default). No fixed random seed was specified. Each report was submitted three consecutive times per model as independent API calls to quantify output stability; intra-model agreement was then calculated based on the three returned TNM outputs. For inter-model agreement, the most frequent prediction across the three runs was considered the model's final output. In cases where all three predictions differed, adjudication was performed by a senior radiologist.

### Human reader evaluation

Five junior radiologists independently assigned T, N, and M categories from the PET-CT report text. All were trained in the AJCC 8th edition criteria but blinded to the reference standard and LLM results. In addition to reader-wise performance, an aggregated junior-radiologist comparator was constructed using majority voting: for each case, the T, N, and M categories were determined separately as the most frequently assigned label across the five readers. In the event of a tie, the reference standard category was used for adjudication. The aggregated TNM stage was considered correct only if all three components (T, N, and M) matched the reference standard. Reading time per case was recorded.

### Statistical analysis

All statistical analyses were performed using Python (version 3.11.3) and SPSS (version 25.0; IBM, Chicago, IL). Model and human reader outputs were compared against the reference standard.

Agreement was evaluated at two levels: intra-model agreement (across three independent runs of each model) using Fleiss' *κ*, and inter-model agreement between different models using Cohen's *κ*. Agreement with junior radiologists was also assessed using Cohen's *κ*, with *κ* values interpreted as slight (≤0.20), fair (0.21–0.40), moderate (0.41–0.60), substantial (0.61–0.80), or almost perfect (≥0.81).

For comparisons of accuracy between LLMs and junior radiologists, the McNemar test was used. Processing times were compared using the Wilcoxon signed-rank test. Receiver operating characteristic (ROC) analysis was performed to calculate area under the curve (AUC) for overall staging accuracy, and DeLong test was applied for AUC comparisons. Subgroup analyses were performed for each cancer type. All statistical tests were two-sided, and a *p* value < 0.05 was considered statistically significant. Bonferroni correction was applied for multiple comparisons.

## Results

### Patient characteristics

A total of 552 patients with pathologically confirmed malignancies were included. The mean age was 58.4 ± 13.2 years (range, 27–86 years), with the majority (53.4%) between 50 and 70 years of age. Demographic and staging characteristics for each cancer type are presented in Table 1.

**Table 1:** Clinical characteristics of patients by cancer type.

Characteristic	Lung cancer (*n* = 118)	Breast cancer (*n* = 96)	Liver cancer (*n* = 102)	Pancreatic cancer (*n* = 72)	Kidney cancer (*n* = 76)	Prostate cancer (*n* = 88)
Age (y)						
Mean ± SD	62.3 ± 9.8	54.7 ± 11.2	58.9 ± 10.5	63.5 ± 8.9	59.2 ± 10.8	65.8 ± 8.2
Range	42–78	32–76	38–75	45–79	35–77	48–82
Age group						
<50	16 (13.6)	24 (25.0)	18 (17.6)	8 (11.1)	14 (18.4)	6 (6.8)
50–70	78 (66.1)	58 (60.4)	64 (62.7)	48 (66.7)	48 (63.2)	54 (61.4)
>70	24 (20.3)	14 (14.6)	20 (19.6)	16 (22.2)	14 (18.4)	28 (31.8)
T stage						
T1	24 (20.3)	20 (20.8)	22 (21.6)	10 (13.9)	18 (23.7)	16 (18.2)
T2	52 (44.1)	44 (45.8)	42 (41.2)	24 (33.3)	30 (39.5)	30 (34.1)
T3	28 (23.7)	22 (22.9)	26 (25.5)	30 (41.7)	18 (23.7)	28 (31.8)
T4	14 (11.9)	10 (10.4)	12 (11.8)	8 (11.1)	10 (13.2)	14 (15.9)
N stage						
N0	30 (25.4)	26 (27.1)	66 (64.7)	16 (22.2)	52 (68.4)	56 (63.6)
N1	46 (39.0)	42 (43.8)	24 (23.5)	38 (52.8)	16 (21.1)	22 (25.0)
N2	28 (23.7)	18 (18.8)	8 (7.8)	12 (16.7)	6 (7.9)	8 (9.1)
N3	14 (11.9)	10 (10.4)	4 (3.9)	6 (8.3)	2 (2.6)	2 (2.3)
M stage						
M0	84 (71.2)	74 (77.1)	84 (82.4)	44 (61.1)	62 (81.6)	72 (81.8)
M1	34 (28.8)	22 (22.9)	18 (17.6)	28 (38.9)	14 (18.4)	16 (18.2)

Data are numbers of findings, with percentages in parentheses.

For lung cancer (*n* = 118), most patients were staged as T2 (0.46), N1 (0.39), and M0 (0.71). For breast cancer (*n* = 96), T2 (0.48), N1 (0.44), and M0 (0.77) were predominant. In hepatocellular carcinoma (*n* = 102), T2 (0.41) was most frequent, with fewer nodal and distant metastases (N0: 0.65; M0: 0.82). Pancreatic cancer (*n* = 72) cases typically presented at more advanced stages, with T3 (0.42), N1 (0.53), and a relatively high proportion of distant metastases (M1: 0.39). For renal cell carcinoma (*n* = 76), early disease was common, with T1 (0.45) and low rates of nodal (N0: 0.68) and distant metastases (M0: 0.82). Prostate cancer (*n* = 88) demonstrated a more balanced distribution, with T2 (0.34) and T3 (0.32) most frequently observed.

### Overall performance of LLMs and junior radiologists

ChatGPT 5 achieved the highest overall accuracy (0.82; 95% CI: 0.79–0.85), followed by ChatGPT 4o (0.74; 95% CI: 0.71–0.78) and ChatGPT 3.5 (0.60; 95% CI: 0.55–0.64). Junior radiologists obtained an accuracy of 0.77 (95% CI: 0.73–0.80). ChatGPT 5 significantly outperformed both ChatGPT 3.5 (*p* < 0.001) and junior radiologists (*p* = 0.041), whereas ChatGPT 4o performed better than ChatGPT 3.5 (*p* < 0.001) but not junior radiologists (*p* = 0.12) (Table 2).

**Table 2:** Overall performance of large language models and junior radiologists.

Model/evaluator	Overall accuracy (95% CI)	Processing time (s)	T Staging accuracy	N Staging accuracy	M Staging accuracy
ChatGPT 5	0.82 (0.79–0.85)	8.3 ± 3.2	0.84 (465/552)	0.79 (436/552)	0.76 (420/552)
ChatGPT 4o	0.74 (0.71–0.78)	7.6 ± 2.9	0.77 (425/552)	0.70 (386/552)	0.67 (370/552)
ChatGPT 3.5	0.60 (0.56–0.64)	12.8 ± 5.1	0.63 (348/552)	0.56 (309/552)	0.53 (292/552)
Junior Radiologists	0.77 (0.73–0.80)	92.5 ± 21.7	0.78 (430/552)	0.72 (397/552)	0.68 (375/552)

Processing time per report was shortest for ChatGPT 5 (8.3 ± 3.2 s) and ChatGPT 4o (7.6 ± 2.9 s), both markedly faster than junior radiologists (92.5 ± 21.7 s; *p* < 0.001). ChatGPT 3.5 required 12.8 ± 5.1 s per case, still significantly faster than humans (*p* < 0.001). Reader-wise performance of the five junior radiologists and the majority-vote aggregate are summarized in Supplementary Table S1.

### Component-level performance

**Figure 2:**
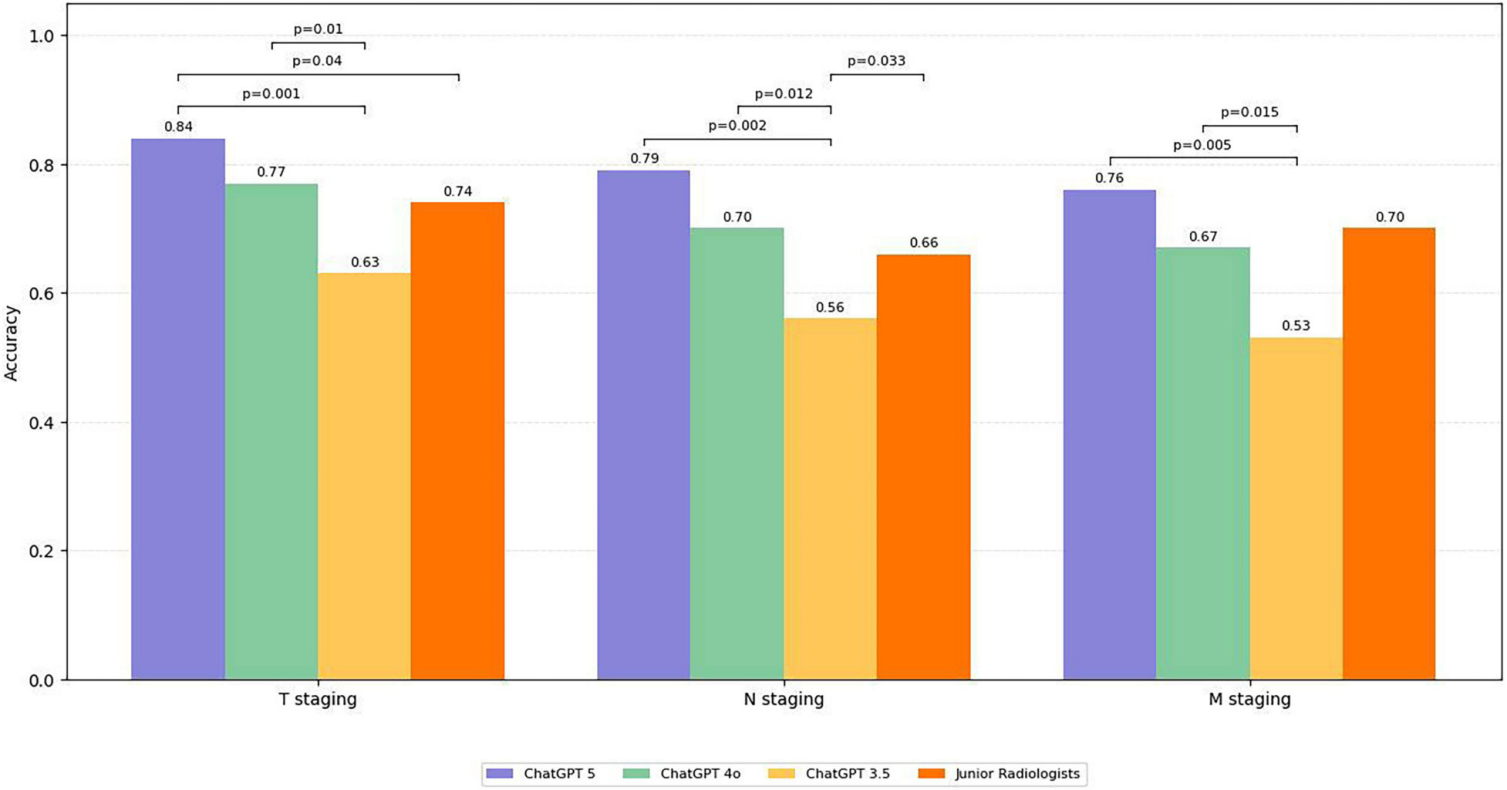
Comparison of staging accuracy by TNM component **(T,N,M)** across three large language models and junior radiologists. ChatGPT 5 consistently achieved the highest accuracy, with T staging showing the best performance across all models. Error bars indicate 95% confidence intervals. Asterisks denote statistically significant differences between models.

Staging accuracy differed by component (Figure 2). T staging showed the highest accuracy across models: ChatGPT-5 0.84 (95% CI: 0.81–0.87), ChatGPT-4o 0.77 (95% CI: 0.73–0.80), and ChatGPT-3.5 0.63 (95% CI: 0.59–0.67). N staging ranked second: ChatGPT-5 0.79 (95% CI: 0.75–0.82), ChatGPT-4o 0.70 (95% CI: 0.66–0.74), and ChatGPT-3.5 0.56 (95% CI: 0.52–0.60). M staging was lowest: ChatGPT-5 0.76 (95% CI: 0.72–0.79), ChatGPT-4o 0.67 (95% CI: 0.63–0.71), and ChatGPT-3.5 0.53 (95% CI: 0.49–0.57). To clarify the sources of misclassification, we quantified component-level error rates for ChatGPT-5 stratified by cancer type (Supplementary Table S2). Across cancer types, errors were more frequently attributable to N- and M-component misclassification than to T-component misclassification.

### Stage-level performance

**Figure 3:**
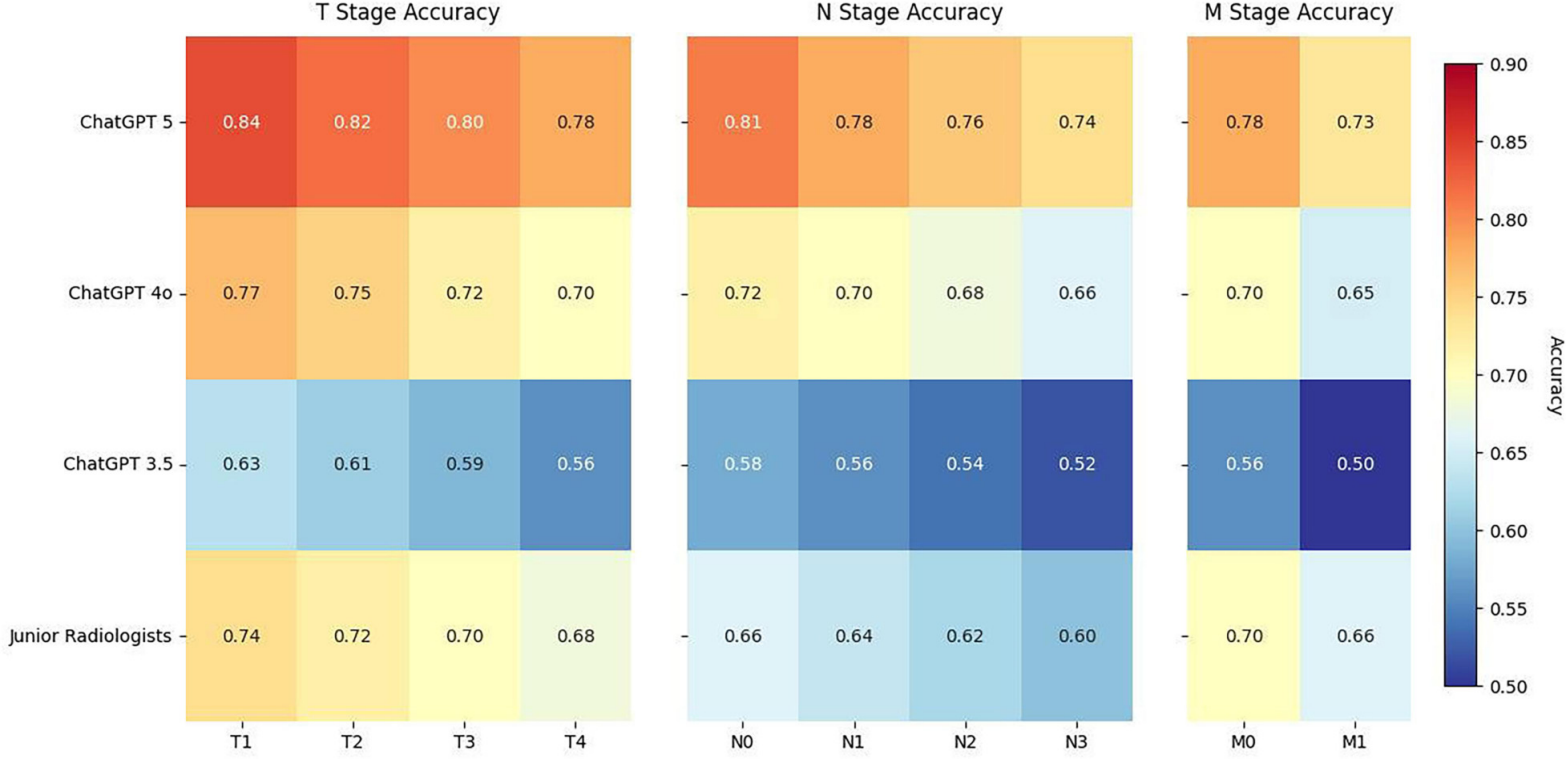
Heatmaps showing classification accuracy by stage subgroup across TNM components and models. Staging accuracy is displayed for T (T1–T4), N (N0–N3), and M (M0–M1) categories. Accuracy declined with increasing stage complexity, particularly for nodal and metastatic disease. ChatGPT 5 and ChatGPT 4o demonstrated higher consistency compared with ChatGPT 3.5 and junior radiologists.

Heatmap analyses further illustrated variation in staging accuracy across subcategories (Figure 3). For T staging, accuracy was highest in early disease (T1–T2) and gradually decreased in advanced stages (T3–T4) across all models. In N staging, recognition of N0 disease was consistently better than higher nodal burden (N2–N3). For M staging, classification of M0 was more reliable than M1, with misclassifications clustering around borderline cases of suspected distant metastases. These trends were consistent across models and aligned with overall component-level accuracy patterns.

### Cancer-specific performance

**Figure 4:**
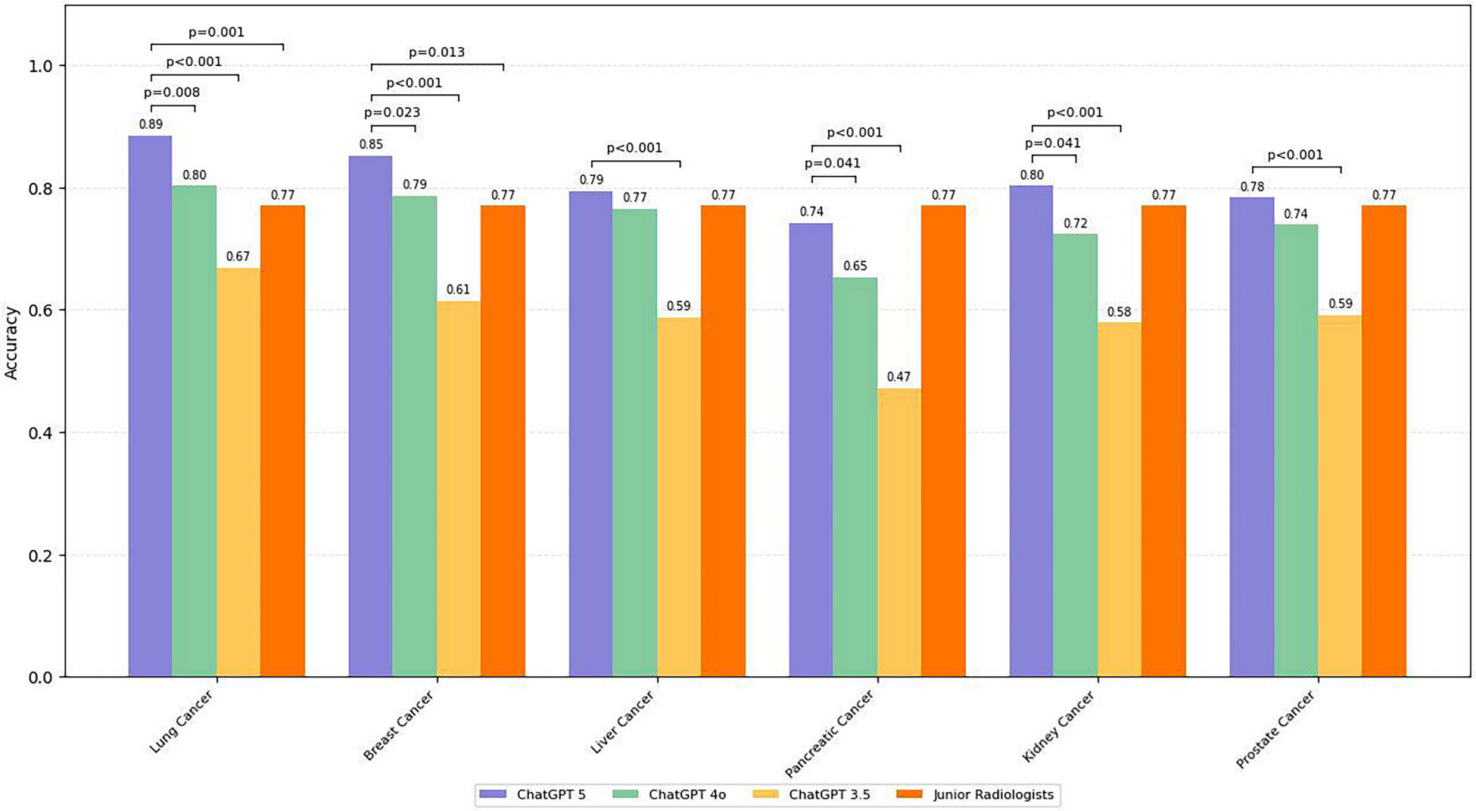
Accuracy of TNM staging by cancer type across large language models and junior radiologists. ChatGPT 5 achieved the highest accuracy in all six malignancies, with lung and breast cancers showing the best performance and pancreatic cancer remaining the most challenging. Statistical significance markers indicate pairwise differences between models.

Accuracy varied by cancer type (Figure 4, Table 3). Lung and breast cancers demonstrated the highest performance, with ChatGPT 5 exceeding 0.85 in both, significantly higher than ChatGPT 3.5 (*p* < 0.01). Liver cancer showed slightly lower performance, with ChatGPT 5 at 0.79 (95% CI: 0.73–0.84) and ChatGPT 4o at 0.77 (95% CI: 0.71–0.82), both outperforming ChatGPT 3.5 (0.59; 95% CI: 0.52–0.65). Pancreatic cancer was the most challenging, with ChatGPT 5 achieving 0.74 (95% CI: 0.66–0.81) and ChatGPT 3.5 dropping below 0.50 (95% CI: 0.41–0.54) (*p* < 0.001). Renal and prostate cancers showed intermediate results, with ChatGPT 5 consistently outperforming ChatGPT 4o by 0.06–0.08, and both models substantially surpassing ChatGPT 3.5 (all *p* < 0.05). Representative correct and misclassified cases with sentence-level evidence citation and component-level error attribution are provided in Supplementary Material I.

**Table 3:** Intra-model and inter-model agreement analysis.

Agreement type	Models/comparison	Kappa (95% CI)
Intra-model Agreement	ChatGPT 5	0.96 (0.93–0.98)
	ChatGPT 4o	0.89 (0.85–0.92)
	ChatGPT 3.5	0.77 (0.71–0.82)
	Junior Radiologists	0.81 (0.77–0.85)
Inter-model Agreement	ChatGPT 5 vs. ChatGPT 4o	0.78 (0.73–0.83)
	ChatGPT 5 vs. ChatGPT 3.5	0.61 (0.56–0.66)
	ChatGPT 4o vs. ChatGPT 3.5	0.58 (0.52–0.63)

Data are *κ* values, with 95% confidence intervals. Intra-LLM agreement was measured using Fleiss *κ*; inter-LLM agreement was measured using Cohen *κ*. Agreement was categorized as slight (≤0.20), fair (0.21–0.40), moderate (0.41–0.60), substantial (0.61–0.80), or almost perfect (0.81–1.00).

### Agreement analysis

Intra-model agreement was almost perfect for ChatGPT 5 (*κ* = 0.96; 95% CI: 0.93–0.98) and ChatGPT 4o (*κ* = 0.89; 95% CI: 0.85–0.92), and substantial for ChatGPT 3.5 (*κ* = 0.77; 95% CI: 0.71–0.82). Inter-model agreement was substantial for ChatGPT 5 vs. ChatGPT 4o (*κ* = 0.78; 95% CI: 0.73–0.83) and ChatGPT 5 vs. ChatGPT 3.5 (*κ* = 0.61; 95% CI: 0.56–0.66), and moderate for ChatGPT 4o vs. ChatGPT 3.5 (*κ* = 0.58; 95% CI: 0.52–0.63). Agreement between junior radiologists and the reference standard was also almost perfect (*κ* = 0.81; 95% CI: 0.77–0.85), although numerically lower than that of ChatGPT 5 and ChatGPT 4o.

## Discussion

This study assessed the ability of three large language models including ChatGPT 5, ChatGPT 4o, and ChatGPT 3.5, to automate TNM staging from PET-CT reports across six common malignancies. By directly comparing different generations of models with junior radiologists, we provide evidence on the evolving role of LLMs in radiologic oncology. The results show that ChatGPT 5 achieved the highest overall accuracy, consistently surpassing ChatGPT 4o and ChatGPT 3.5, and outperforming junior radiologists in both staging accuracy and intra-observer consistency. These findings suggest that the newest generation of models may be approaching the reliability needed for clinical applications where structured interpretation of free-text reports is required.

Earlier studies of ChatGPT in radiology have mostly emphasized report structuring, protocol selection, or answering board-style questions (12–14). Few have examined its performance on systematic staging, a task that requires not only recognition of keywords but also integration of scattered observations into codified staging rules. Our results indicate that newer model generations can more reliably perform report-to-stage mapping under AJCC criteria at the case level. The contrast between ChatGPT 5 and its predecessors underscores the rapid progress in LLM capabilities, with accuracy gains evident across nearly all cancer types and staging components. In this regard, the current findings extend previous work by showing that LLMs are not limited to impression generation but can also support more logic-driven clinical tasks (15, 16).

Differences across staging components provide useful insight into where LLMs succeed and where they struggle. T staging achieved the highest accuracy, reflecting the relatively standardized language used to describe tumor size and local invasion (17). Report phrases such as “mass measuring 3.5 cm” or “extension into chest wall” are clear and map directly to staging thresholds, allowing even earlier models to perform reasonably well. In contrast, N staging posed greater challenges. Lymph node involvement is often described with variability and uncertainty, such as “borderline enlarged” or “suspicious for metastasis,” making consistent interpretation more difficult (18). This explains the lower accuracy across all models and highlights an area where automated systems may need additional refinement. M staging showed mixed results. Negative statements about distant disease were generally interpreted correctly, but scattered positive findings, particularly when presented with cautionary wording, were frequently misclassified (19). This mirrors the difficulties faced by less experienced radiologists, emphasizing that some challenges are inherent to the task itself rather than to the model alone.

Performance also varied by cancer type. Lung and breast cancers achieved the highest accuracy, likely reflecting the fact that their staging criteria are well standardized and frequently encountered in radiology reports used to train LLMs (20). Pancreatic cancer, by contrast, proved the most difficult. This is consistent with clinical experience, as pancreatic tumors often involve subtle findings such as vascular encasement or peritoneal spread that may not be consistently described in text (21). Intermediate results were seen for liver, kidney, and prostate cancers, again suggesting that the clarity and consistency of reporting language strongly influence model performance. These differences highlight the dual dependency of automated staging on both model reasoning and the structure of the source reports.

Reliability is as important as accuracy in any tool intended for clinical decision support. Intra-model agreement was strongest for ChatGPT 5, approaching the threshold of “almost perfect” agreement, while ChatGPT 4o and ChatGPT 3.5 showed progressively lower consistency (22). The stability of outputs across repeated runs is reassuring, given concerns about stochasticity in generative models (23). Interestingly, junior radiologists demonstrated lower repeatability than ChatGPT 5, underscoring that human interpretation is not free from variability even in structured tasks (24). These findings suggest that advanced LLMs may contribute not only speed but also consistency, potentially reducing inter-reader variability that has long challenged oncologic staging (25).

The clinical consequences of TNM mis-staging warrant careful consideration before deployment. Errors in N or M classification may carry disproportionate downstream impact, including inappropriate treatment intensity, incorrect eligibility assessment for clinical trials, and flawed longitudinal comparisons. These risks may be amplified by ambiguity or incomplete specification in narrative reports, which can complicate consistent mapping to AJCC staging rules. Accordingly, outputs from LLMs should be treated as decision-support suggestions rather than definitive staging. Practical guardrails can mitigate these risks by limiting use to human-verified decision support, requiring sentence-level evidence citation for each TNM component, automatically flagging uncertain or internally discordant outputs for senior review, and preserving an audit trail for quality assurance and governance.

Automated staging could provide rapid preliminary classifications, assisting junior radiologists in daily practice and accelerating preparation for multidisciplinary tumor boards (26). The substantial time savings observed with ChatGPT models compared with human readers further support their potential role in workflow optimization (27). In a practical workflow, the model could be deployed after report finalization to pre-populate structured TNM fields with sentence-level evidence from the report for rapid human verification before tumor board submission. This human-in-the-loop design preserves clinical accountability while leveraging the observed time savings and improving staging documentation consistency. More broadly, the ability to extract structured staging data from large numbers of free-text reports may facilitate clinical research, registry development, and quality monitoring (28). Importantly, these models are best conceived as augmentative rather than replacement tools. Their outputs can serve as staging suggestions that require expert confirmation, fitting naturally into the paradigm of augmented intelligence in radiology (29).

Our study has several limitations. The sample size, while spanning six malignancies, still provided limited representation of advanced stages such as T4 or N3 disease, which may restrict generalizability for these categories. The retrospective design relied on existing reports, and subtle features influencing staging could have been omitted or inconsistently described. All data were in English and derived from two institutions, which limits applicability to multilingual and more diverse clinical environments. Furthermore, we evaluated only one LLM family (ChatGPT) and did not benchmark against other competitive general-purpose or medical-oriented models. As a result, the absolute performance reported here may not directly translate to models with different training corpora, safety constraints, or inference settings, and our conclusions should be interpreted primarily as comparative evidence across ChatGPT generations. The models were tested through API calls without domain-specific fine-tuning, mirroring real-world use but leaving open the possibility of improved performance with adaptation. Finally, staging was assessed in isolation, whereas in clinical workflows decisions are integrated with pathology, laboratory, and clinical data. Future prospective evaluations in routine practice will be necessary before clinical deployment.

In conclusion, this study demonstrates that LLMs, and particularly ChatGPT 5, can achieve reliable performance in TNM staging from PET-CT reports across multiple cancer types. Accuracy was highest for T staging and lowest for nodal assessment, with clear differences across malignancies. Compared with junior radiologists, the newest model showed both higher accuracy and greater consistency, while processing cases substantially faster. Although limitations remain, these results suggest that LLMs are approaching a level of performance that could make them valuable as adjunctive tools in oncologic imaging, capable of enhancing efficiency, standardization, and reproducibility in cancer staging.

## Data Availability

The original contributions presented in the study are included in the article/Supplementary Material, further inquiries can be directed to the corresponding author.
