# Supplementary material for: Evaluating large language models for automated TNM staging from PET-CT reports: a multi-cancer comparative study
Source: Front Digit Health. 2026 Mar 4;8:1741973. doi: 10.3389/fdgth.2026.1741973 (PMC12996206; doi:10.3389/fdgth.2026.1741973)
Supplement: Supplementary file 1 [file Table1.docx]

**Supplementary Material I**

**Representative de-identified PET-CT report input, Lung cancer**

**Model input：**

**FINDINGS:**

Brain: Normal brain uptake pattern without significant abnormalities. Midline structures are centered. No ventricular enlargement or widening/deepening of cerebral sulci. Cerebellum shows normal uptake with symmetrical hemispheres. Head and Neck: Bilateral inferior turbinate hypertrophy with mild radiotracer uptake (SUVmax 1.94). No significant nasopharyngeal thickening; bilateral pharyngeal recess shows increased uptake (SUVmax 4.13). Bilateral oropharyngeal walls and base of tongue appear slightly prominent with increased uptake (SUVmax 4.3). Thyroid glands show normal morphology with heterogeneous density, no abnormal uptake. Left cervical vascular space and left submandibular region show nodules, largest measuring 10x8mm with CT value of 59HU and increased uptake (SUVmax 3.73). Multiple small nodes (<6mm) in bilateral neck and supraclavicular regions with mild uptake (SUVmax 2.35). Remaining maxillofacial and cervical structures show normal morphology and radiotracer distribution. Chest: Left upper lobe lingular segment shows an irregular mass with lobulated margins, unclear boundary with oblique fissure, adjacent pleural thickening and retraction, associated bronchial tail sign, measuring 36x33x46mm, CT value 30-43HU, with increased uptake (SUVmax 8.47). Multiple bilateral pulmonary micronodules <3mm without significant uptake. Left lower lobe posterior basal segment shows a micronodule <3mm. Multiple small nodes in mediastinum (peritectal, retro-SVC, main pulmonary window, subcarinal, cardiophrenic, right costophrenic regions), largest 7mm with mild uptake (SUVmax 1.97). No enlarged hilar lymph nodes or abnormal uptake. Focal bilateral pleural thickening without abnormal uptake. No pleural effusion. Normal cardiac and great vessel uptake. Symmetric breast tissue without abnormal uptake. No enlarged axillary nodes or abnormal uptake. Abdomen: Liver shows normal morphology with smooth contours and normal lobar proportions. Small round hypodense lesion in left lobe (6x5mm) without abnormal uptake. Normal gallbladder size with homogeneous density, no wall thickening, contains laminated high-density material, no abnormal uptake. Pancreas shows normal morphology and uptake, no ductal dilation. Spleen normal in size with uniform uptake. Bilateral kidneys normal with homogeneous parenchymal density, no abnormal uptake. No hydronephrosis or hydroureter. Adrenal glands grossly normal without significant uptake. Normal gastric and duodenal uptake. Multiple bowel loops of varying density and morphology. No enlarged abdominal or retroperitoneal lymph nodes or abnormal uptake. No ascites. Pelvis: Normal bladder uptake without wall thickening. No enlarged lymph nodes or abnormal uptake in bilateral iliac and inguinal regions. Uterus shows focal protrusion without abnormal uptake. Central uterine hypodensity with focal uptake (SUVmax 4.29). Bilateral adnexal regions appear prominent with increased uptake (SUVmax 4.61). Pelvic fluid collection with mild uptake (SUVmax 1.94). Skeletal System: Increased uptake noted in atlas vertebra, right scapula, right transverse process of T9, right lateral process of L1, sacrum, left ilium, and left acetabulum (SUVmax 8.44) with corresponding CT hypodensities or heterogeneous density. Straightening of cervical lordosis. Otherwise normal skeletal and joint morphology, density, and uptake distribution.

**IMPRESSION:**

1. Left upper lobe hypermetabolic mass, highly suspicious for lung cancer; biopsy recommended. 2. Multiple hypermetabolic osseous lesions with corresponding CT changes, consistent with skeletal metastases. 3. Left cervical and submandibular nodes with moderate to high metabolic activity, suspicious for lymph node metastases; close monitoring required. 4. Multiple small mediastinal nodes with mild uptake, likely representing inflammatory/reactive changes; observation recommended. 5. Multiple bilateral pulmonary micronodules without significant uptake, likely inflammatory granulomas; periodic follow-up advised. 6. Bilateral inferior turbinate hypertrophy; nasopharyngeal uptake likely inflammatory. 7. Heterogeneous thyroid density without abnormal uptake, likely benign; ultrasound correlation recommended. 8. Left lower lobe microcalcification; bilateral focal pleural thickening. 9. Hepatic cyst; gallbladder sludge, correlate clinically. 10. Uterine protrusion without abnormal uptake, possible leiomyoma; central uterine hypodensity with focal uptake and bilateral adnexal uptake likely physiologic; correlate with menstrual history and ultrasound. 11. Minimal pelvic fluid. 12. Cervical spondylosis. No other evidence of malignancy on whole-body PET-CT imaging.

**Reference standard** (cTNM, AJCC 8th): T2b N0 M1c

**Model output**: a correct staging example and two representative failure modes

Model and run: ChatGPT 5 (Run 1)

Model output TNM: T2b N0 M1c

Correct overall?: Yes

Error component(s): None

Evidence sentences from report:

T: “Irregular mass… measuring 36×33×46 mm… SUVmax 8.47.”

N: “Mediastinal nodes… largest 7 mm with mild uptake (SUVmax 1.97)… likely inflammatory/reactive.”

M: “Multiple hypermetabolic osseous lesions… consistent with skeletal metastases.”

Brief rationale (evidence-linked):

Maximal diameter 46 mm maps to lung cancer T2b; nodal descriptions remain subcentimeter with reactive impression → N0; multiple bone metastases → M1c.

Model: ChatGPT 4o (Run 1)

Model output TNM: T2b N3 M1c

Correct overall?: No

Error component(s): N

Evidence sentences from report:

T: “Mass… 36×33×46 mm…”

N (misleading cue): “Left cervical vascular space and left submandibular region show nodules… increased uptake (SUVmax 3.73)… Impression: suspicious for lymph node metastases.”

N (counter-evidence): “Mediastinal nodes… mild uptake… likely inflammatory/reactive; no enlarged hilar lymph nodes.”

M: “Multiple hypermetabolic osseous lesions… skeletal metastases.”

Brief rationale (evidence-linked):

The model may over-weight “cervical/submandibular nodes… suspicious” and incorrectly map non-regional nodes to regional N3 rather than treating them as distant disease context or as non-definitive for regional nodal staging in lung cancer.

Model: ChatGPT 3.5 (Run 1)

Model output TNM: T2b N3 M1b

Correct overall?: No

Error component(s): N, M

Evidence sentences from report:

T: “Left upper lobe lingular segment shows an irregular mass… measuring 36×33×46 mm… SUVmax 8.47.”

N (misleading cue): “Left cervical vascular space and left submandibular region show nodules… increased uptake (SUVmax 3.73)… Impression: suspicious for lymph node metastases.”

N (counter-evidence): “Multiple small nodes in mediastinum… largest 7 mm with mild uptake (SUVmax 1.97)… Impression: likely inflammatory/reactive; no enlarged hilar lymph nodes.”

M: “Multiple hypermetabolic osseous lesions… consistent with skeletal metastases.”

Brief rationale (evidence-linked):

The model may over-weight the impression statement “suspicious for lymph node metastases” involving cervical/submandibular nodes and incorrectly map these non-thoracic nodes to regional N3, despite counter-evidence suggesting thoracic nodes are reactive. It may also recognize metastatic disease but misapply AJCC subcategorization, collapsing “multiple extrathoracic skeletal metastases” into M1b rather than M1c, producing simultaneous N and M misclassification while retaining the correct size-based T2b assignment.

**Supplementary Material II：Supplementary Tables**

**Table S1. Reader-wise and majority-vote performance of junior radiologists (N=552)**

| **Metric** | **Reader 1** | **Reader 2** | **Reader 3** | **Reader 4** | **Reader 5** | **Mean**  **(SD)** | **Majority vote** |
| --- | --- | --- | --- | --- | --- | --- | --- |
| Overall TNM accuracy, n/N (proportion; 95% CI) | 418/552 (0.76; 0.72–0.79) | 426/552 (0.77; 0.73–0.80) | 410/552 (0.74; 0.70–0.78) | 432/552 (0.78; 0.75–0.82) | 421/552 (0.76; 0.73–0.80) | 0.76 (0.01) | 425/552 (0.77;0.73–0.80) |
| T accuracy, n/N (proportion; 95% CI) | 425/552 (0.77; 0.73–0.80) | 433/552 (0.78; 0.75–0.82) | 420/552 (0.76; 0.72–0.79) | 438/552 (0.79; 0.76–0.83) | 429/552 (0.78; 0.74–0.81) | 0.78 (0.01) | 430/552 (0.78;0.74–0.81) |
| N accuracy, n/N (proportion; 95% CI) | 392/552 (0.71; 0.67–0.75) | 401/552 (0.73; 0.69–0.76) | 385/552 (0.70; 0.66–0.73) | 410/552 (0.74; 0.70–0.78) | 398/552 (0.72; 0.68–0.76) | 0.72 (0.02) | 397/552 (0.72;0.68–0.76) |
| M accuracy, n/N (proportion; 95% CI) | 368/552 (0.67; 0.63–0.70) | 380/552 (0.69; 0.65–0.73) | 360/552 (0.65; 0.61–0.69) | 388/552 (0.70; 0.66–0.74) | 379/552 (0.69; 0.65–0.72) | 0.68 (0.02) | 375/552 (0.68;0.64–0.72) |
| Agreement with reference (Cohen κ), overall TNM (95% CI) | 0.79 (0.75–0.83) | 0.81 (0.77–0.85) | 0.77 (0.73–0.81) | 0.82 (0.78–0.86) | 0.80 (0.76–0.84) | 0.80 (0.02) | 0.81 (0.77–0.85) |

**Table S2. Component-wise error rates by cancer type for ChatGPT-5**

| Cancer type | n | T incorrect | N incorrect | M incorrect |
| --- | --- | --- | --- | --- |
| Lung cancer | 118 | 12/118  (0.10; 95% CI 0.06–0.17) | 20/118  (0.17; 95% CI 0.11–0.25) | 22/118  (0.19; 95% CI 0.13–0.27) |
| Breast cancer | 96 | 13/96  (0.14; 95% CI 0.08–0.22) | 22/96  (0.23; 95% CI 0.16–0.32) | 24/96  (0.25; 95% CI 0.17–0.35) |
| Liver cancer | 102 | 16/102  (0.16; 95% CI 0.10–0.24) | 18/102  (0.18; 95% CI 0.11–0.26) | 28/102  (0.27; 95% CI 0.20–0.37) |
| Pancreatic cancer | 72 | 20/72  (0.28; 95% CI 0.19–0.39) | 22/72  (0.31; 95% CI 0.21–0.42) | 24/72  (0.33; 95% CI 0.24–0.45) |
| Kidney cancer | 76 | 10/76  (0.13; 95% CI 0.07–0.23) | 14/76  (0.18; 95% CI 0.11–0.29) | 16/76  (0.21; 95% CI 0.13–0.31) |
| Prostate cancer | 88 | 17/88  (0.19; 95% CI 0.12–0.29) | 20/88  (0.23; 95% CI 0.15–0.33) | 18/88  (0.20; 95% CI 0.13–0.30) |
| Total | 552 | 88/552  (0.16; 95% CI 0.13–0.19) | 116/552  (0.21; 95% CI 0.18–0.25) | 132/552  (0.24; 95% CI 0.21–0.28) |
